# Fetal malnutrition-induced catch up failure is caused by elevated levels of miR-322 in rats

**DOI:** 10.1038/s41598-020-58392-x

**Published:** 2020-01-28

**Authors:** Takahiro Nemoto, Yoshihiko Kakinuma

**Affiliations:** Department of Physiology, Nippon Medical School 1-1-5 Sendagi, Bunkyo-ku, Tokyo 113-8602 Japan

**Keywords:** Disease model, Diagnostic markers, Growth disorders, Molecular medicine

## Abstract

If sufficient nutrition is not obtained during pregnancy, the fetus changes its endocrine system and metabolism to protect the brain, resulting in a loss of body size. The detailed mechanisms that determine the success or failure of growth catch-up are still unknown. Therefore, we investigated the mechanism by which catch-up growth failure occurs. The body weights of rat pups at birth from dams whose calorie intake during pregnancy was reduced by 40% were significantly lower than those of controls, and some offspring failed to catch up. Short-body-length and low-bodyweight rats showed blood IGF-1 levels and mRNA expression levels of IGF-1 and growth hormone receptor (GHR) in the liver that were lower than those in controls. The next generation offspring from low-bodyweight non-catch-up (LBW-NCG) rats had high expression of miR-322 and low expression of GHR and IGF-1. The expression of miR-322 showed a significant negative correlation with GHR expression and body length, and overexpression of miR-322 suppressed GHR expression. We found that insufficient intake of calories during pregnancy causes catch-up growth failure due to increased expression of miR-322 and decreased expression of GHR in the livers of offspring, and this effect is inherited by the next generation.

## Introduction

According to the theory of Developmental Origins of Health and Disease (DOHaD)^1,2^, low-birthweight infants are considered to be at risk for noncommunicable chronic diseases (NCDs). Intrauterine nutrition due to insufficient nutrition intake of pregnant mothers results in a fetus having a ‘thrifty’ phenotype^3^. If sufficient nutrition is not supplied from the mother’s body, the fetus will change its own metabolic/endocrine system to maintain brain weight, and as a trade-off will survive by having a low birth weight. If the environment after birth is one of poor nutrition similar to that in the uterus, the phenotype works effectively and the influence on health is small. However, these changes in the metabolism/endocrine system are disadvantageous for adapting to a nutrient-rich environment after birth, and a mismatch between phenotype and environment is likely to occur, resulting a risk of increased NCDs after growth^4^. In a Dutch famine cohort study, the children of pregnant women who were malnourished during their first trimester of pregnancy showed not only increased rates of diabetes, lipid metabolic disorders, obesity, hypertension, and cardiovascular disease, but also stress-related disorders and cognitive dysfunction, including depression^5–13^. It has also been reported that low-birthweight infants may have social and emotional problems and are at risk for psychological disorders. In Japan, an increase in the rate of low-birthweight infants and a decrease in average birth weight have been reported, and there is concern that the average height of Japanese will decrease in the future^14^. Thus, countermeasures against the increase in low-birthweight infants are necessary in Japan.

The cause of low birth weight is intrauterine growth retardation (IUGR) and premature birth. Intrauterine growth retardation is caused by genetic factors, placenta/fetal factors, and maternal factors^15^. As mentioned above, in Japan, low-weight births due to a lack of caloric intake by the mother have increased despite this being a preventable factor. Many low-birthweight infants caused by IUGR catch up to the same heights as healthy children, but some of them will continue to have delayed growth and their final heights will remain smaller than those of healthy children. The details of the endocrinological mechanism of catch-up growth are still unknown. Therefore, we examined the factors involved in catch-up growth in low-birthweight rats, focusing on the growth hormone signaling in the liver, since it is thought that animals maintain brain weight and reduce body weight to cope with uterine undernutrition according to the ‘thrifty’ phenotype theory^4^. We hypothesized that not only would there be a decrease in GH receptor expression level in the liver, but also some additional mechanism responsible for a reduction of growth hormone signaling. One possible mechanism is the regulation of gene expression by microRNA (miRNA). In this study, we identified miR-322 as a microRNA that suppresses liver GHR. It has been reported that miR-322 is expressed not only in the liver, but also in many tissues such as skeletal muscle^16^, chondrocytes^17^, and vascular smooth muscle^18^. Previous studies have reported that miR-322 reduces IGF-1 and IGF1R expression in several tissues^19–21^. We further found in this study that low birth weight transmits between generations and hypothesized that microRNAs are involved in the mechanism of intergenerational transmission. A direct causal relationship between miRNA and DNA methylation is unknown, but the importance of miRNA as a gene expression regulatory factor has been thoroughly demonstrated^22^. A previous cohort study showed that maternal caloric deficiency also affects children of the next generation^23^, so we hypothesized that epigenetic modifications were affecting offspring growth. Epigenetics is defined as mitotically and meiotically heritable changes in gene expression that do not involve a change in the DNA sequence. Two major areas of epigenetics—DNA methylation and histone modification—are known to have profound effects on gene expression^24^.

In this study, to clarify the growth disorder mechanism of low-birthweight rat offspring caused by carbohydrate and calorie restriction during pregnancy, we studied the expression of miRNAs related to GH receptor expression in the liver and their influence on birth weight in the next generation.

## Results

### Non-catch-up growth in offspring caused by calorie restriction during pregnancy

**Figure 1:**
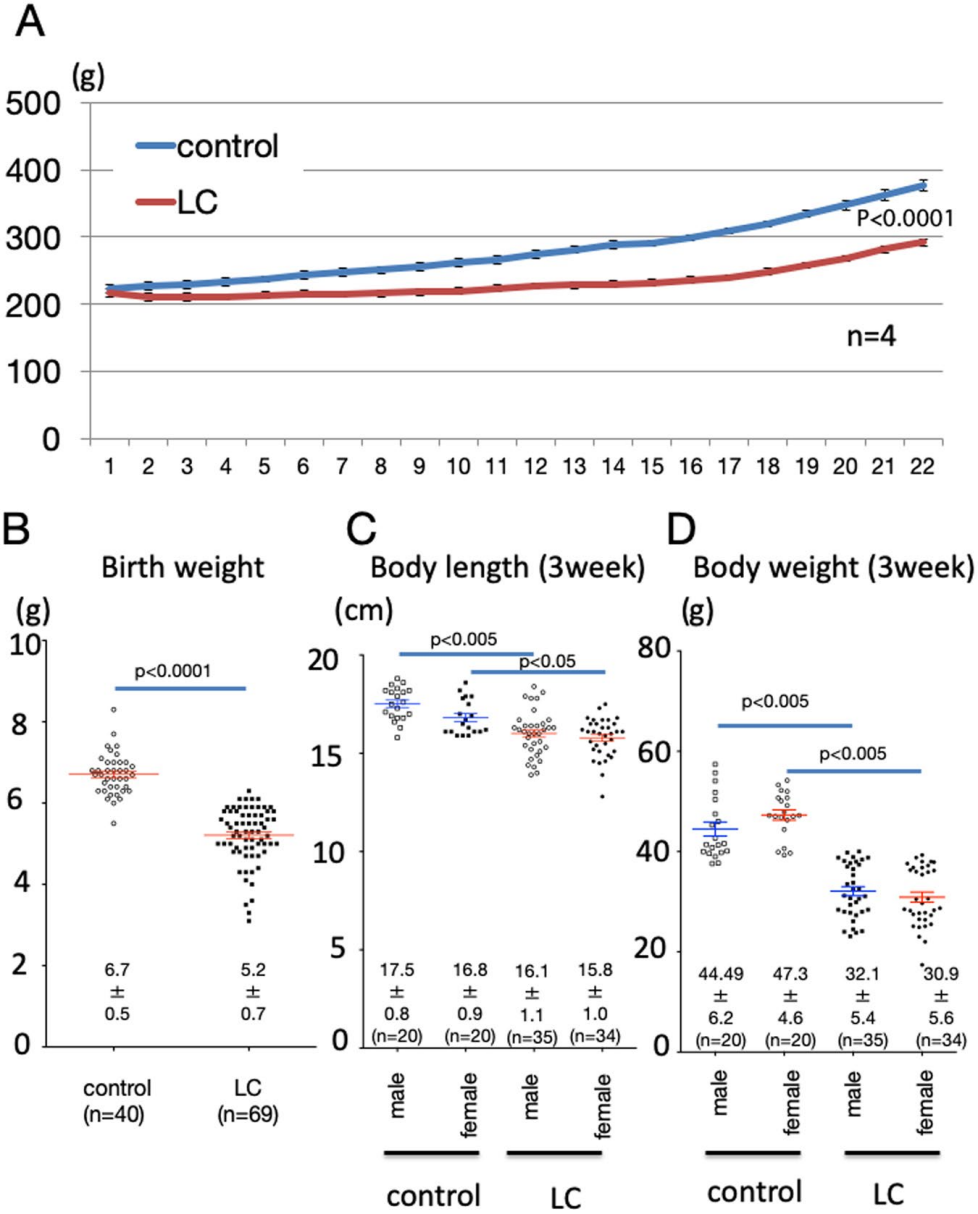
Changes in maternal rat weight due to calorie restriction during pregnancy and the weight of offspring. (**A**) Changes in body weights of mother rats fed a low-carbohydrate, calorie-restricted diet throughout pregnancy. (**B**) Comparison of bodyweights of rat offspring on the day of birth. (**C,D**), Body length and weight at the weaning day. LC indicates low carbohydrate, calorie-restricted mother rats and their offspring. Paired *t* test (**A**) and unpaired *t* tests (**B**–**D**) were used. Data shown are means ± SEM.

The growth of mother rats was significantly suppressed as shown in Fig. 1A due to a low carbohydrate (LC) diet with 40% calorie restriction over the entire gestation period (p < 0.0001, t = 8.384, df = 20). There were no differences in the gestational times of the offspring or the numbers of births, but birth weight was significantly lower for LC mothers than for control mother rats (Fig. 1B). There was no difference in the sex ratio of the offspring. The number of pups was adjusted to 12 per mother rat, and the mother rats after birth were given standard chow by free feeding in both groups. However, some offspring from the caloric-restricted dams failed to catch up in their growth, resulting in short body lengths and low body weights at the weaning day (Fig. 1C,D). There were 15 rats among the 64 LC rats whose growth failed to catch up to the mean within two standard deviations of control rats. We named the groups LC-CG, rat offspring whose growth had caught up, and LC-NCG, rat offspring whose growth did not catch up.

### Endocrinology of LC-NCG offspring

**Figure 2:**
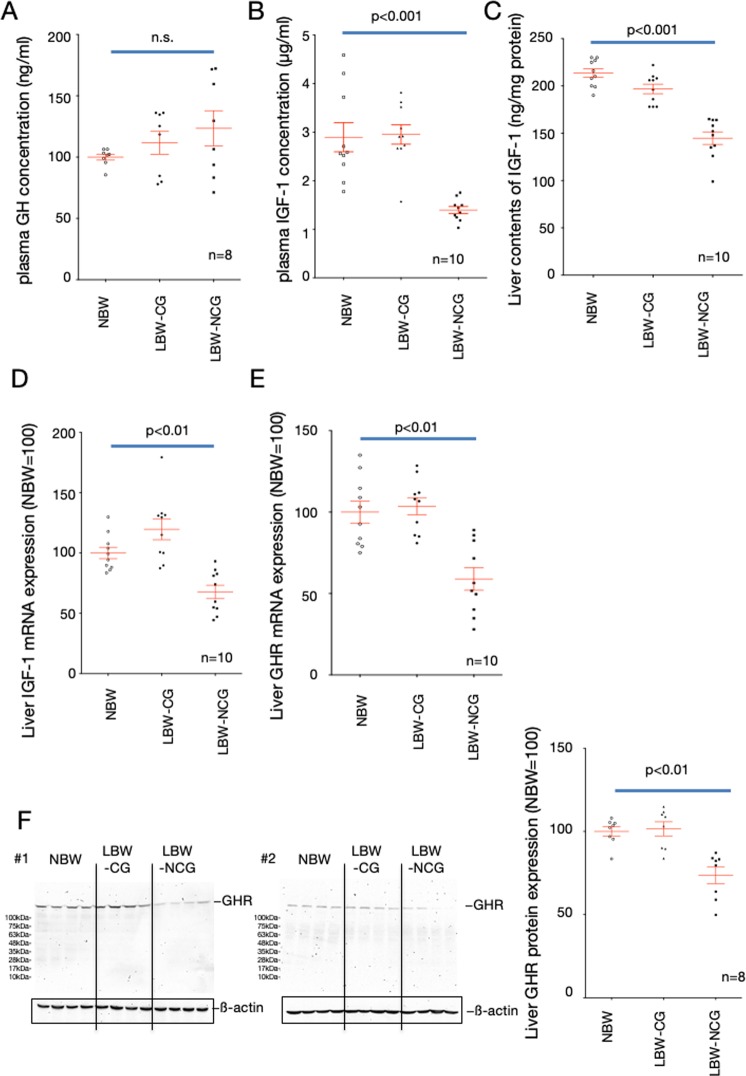
Comparison of hormones in blood and liver of female rats on the weaning day. Concentrations of blood GH (**A**), blood IGF-1 (**B**), tissue IGF-1 (**C**), mRNA expression levels of IGF-1 (**D**) and GH receptor (**E**), and protein expression of GHR (**F**) in livers of female rats at weaning day were quantified. To normalize each sample for RNA and protein content, GAPDH and beta actin were used, respectively. NBW, control rats (normal birth weight); LBW-CG, catch-up growth offspring from low carbohydrate, calorie-restricted dams (low birth weight); LBW-NCG, non-catch-up growth offspring from low carbohydrate, calorie-restricted dams. One-way ANOVA followed by Turkey’s *post hoc* test was used. Data shown are means ± SEM.

There were no statistical differences among control, LC-CG, and LC-NCG rats in measured blood growth hormone (GH) concentrations (Fig. 2A), but blood IGF-1 concentrations and the tissue content of IGF-1 in the livers of LC-NCG were significantly lower than those in the others at weaning day (Fig. 2B,C). The mRNA expression level of IGF-1 in the liver was also significantly lower in LC-NCG compared with control and LC-CG rats (Fig. 2D). To clarify why the concentration and expression of IGF-1 were decreased despite there being no difference in blood GH concentrations in LC-NCG, we next examined the expression levels of GH receptor in the liver. The mRNA and protein expression levels of GH receptor in the livers of LC-NCG rats were significantly lower than those of control and LC-CG rats (Fig. 2E,F).

### Decrease in GH receptor expression level via miRNA

**Figure 3:**
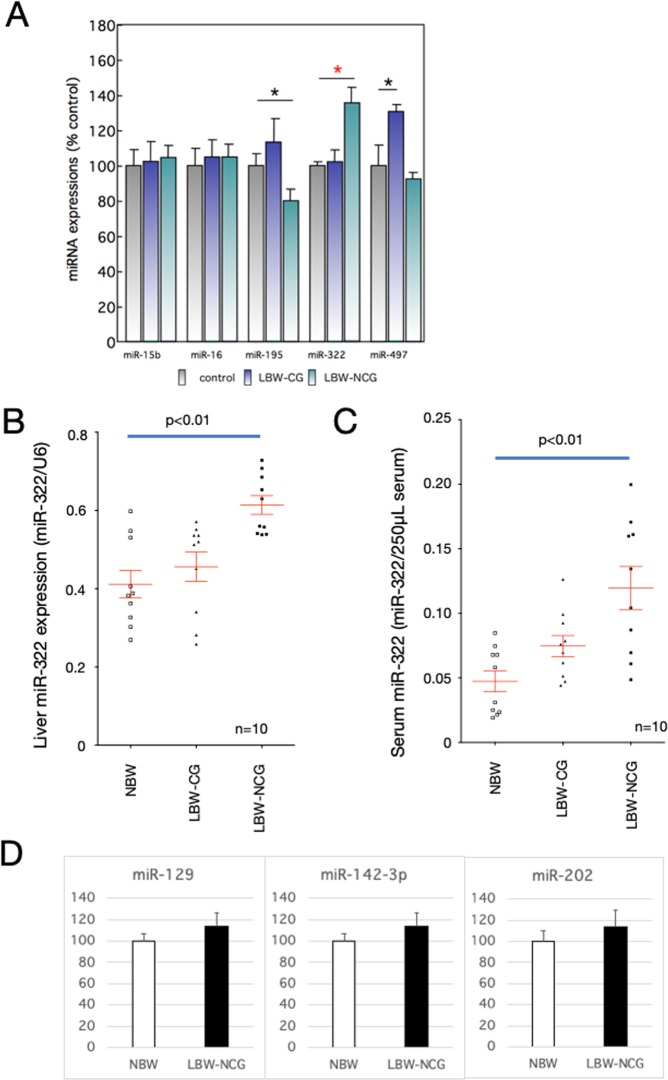
Quantification of miR-322 in liver and blood exosomes. (**A**) All five miRNAs (miR-15b, miR-16, miR-195, miR-322, and miR-497) were quantified from RNA extracted from the livers of rat offspring on the day of weaning (n = 5). *Indicates p < 0.05. (**B**) miR-322 was quantified from RNA extracted from the livers of female rat offspring on the day of weaning (n = 10). (**C**) exosomes were extracted from serum of weaning rats, and miR-322 in the exosomes was quantified (n = 10). (**D**) Other microRNAs (miR-192, miR-142-3p, and miR-2020) were quantified from RNA extracted from the livers of rat offspring on the day of weaning (n = 5). To normalize each sample for RNA content, U6 snRNA was used. NBW, a control rat (normal birth weight); LBW-CG, catch-up growth offspring from low carbohydrate, calorie-restricted dams (low birth weight); LBW-NCG, non-catch-up growth offspring from low carbohydrate, calorie-restricted dams. Unpaired *t* tests were used. Data shown are means ± SEM.

We hypothesized that miRNA might be involved in the decrease in expression levels of GH receptor in the livers of LC-NCG rats. We found five miRNAs with sequences that could bind to the 3′-UTR of the GH receptor in a TargetScan database search. We found that all five miRNA were expressed in the liver. The expression of miR-322 in LC-NCG was higher than that in the other two groups, while the expression of other miRNAs did not differ between the three groups (Fig. 3A,B). As miRNAs are released outside of the cell by exosomes, we collected exosomes in the blood and quantified the miR-322 content. We found that the miR-322 content in blood exosomes in LC-NCG was higher than that in the others (Fig. 3C). There was no significant difference in the expression of other microRNAs that regulate the expression of growth hormone receptors between NBW and LBW-NCG (Fig. 3D).

### The impact of low birth weight on the next generation

**Figure 4:**
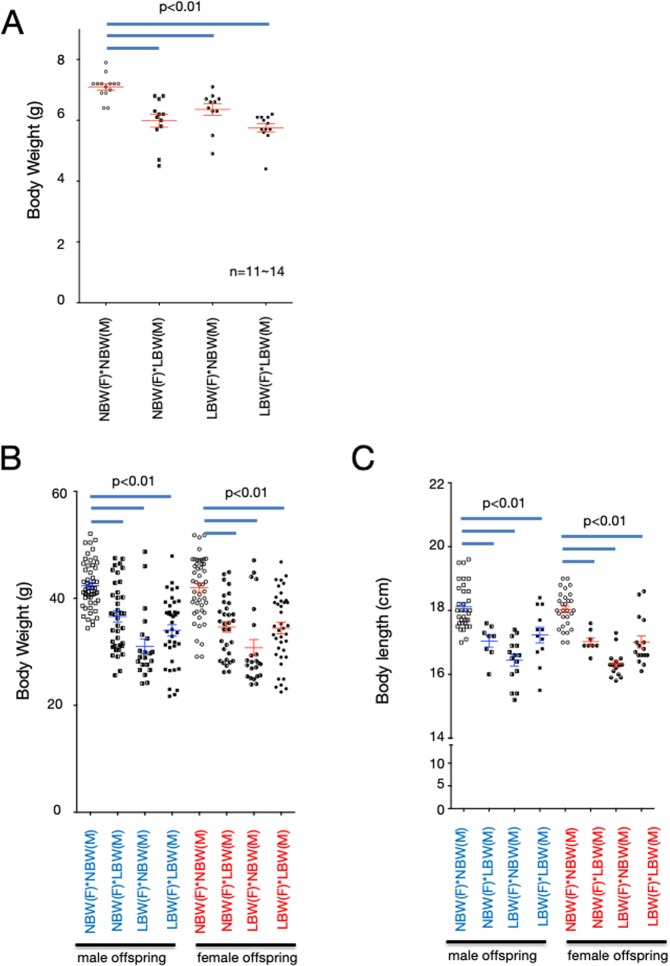
Birth weight and body length and body weight at the weaning day of next-generation offspring. Low-birthweight and non-catch-up growth rats from low carbohydrate, calorie-restricted dams were mated as fathers and/or mothers. Pregnant dams were fed a standard diet *ad libitum*. Body weights at the day of birth (**A**), and body lengths (**B**) and body weights (**C**) at the weaning day of their offspring were measured. One-way ANOVA followed by Turkey’s *post hoc* test was used. Data shown are means ± SEM.

**Figure 5:**
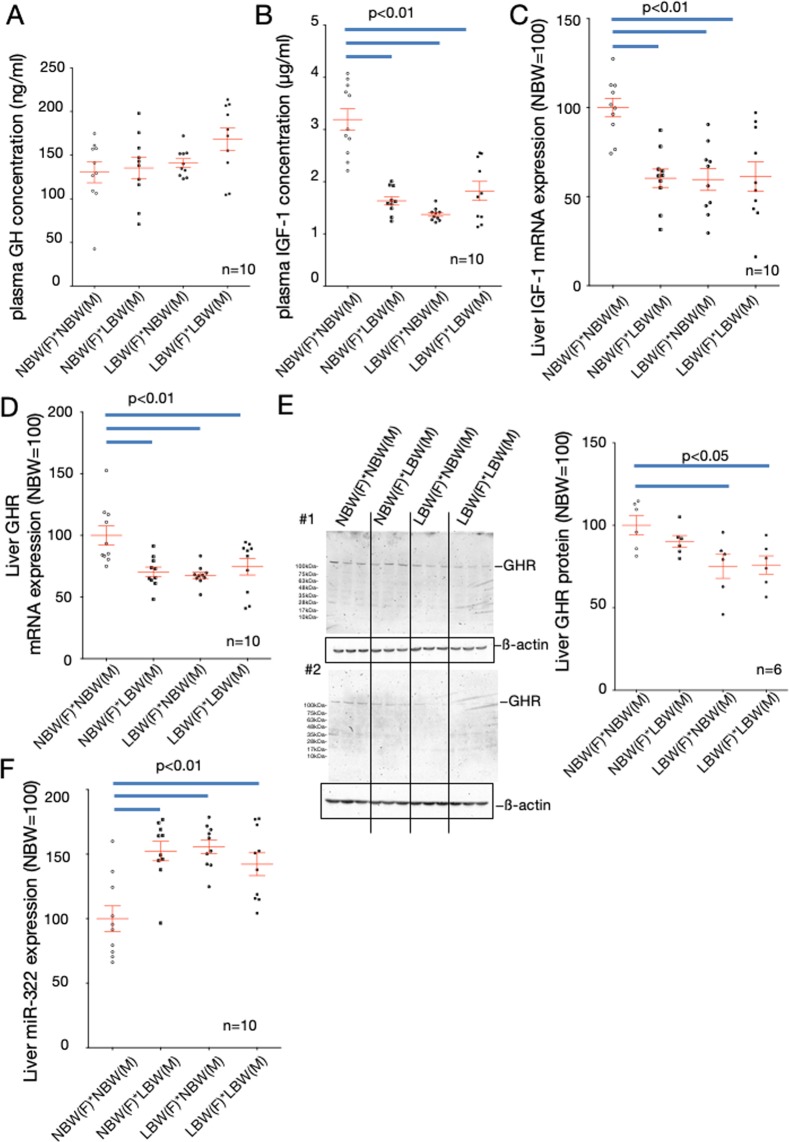
Analysis of blood hormone levels and gene expression in the livers of next-generation offspring. Blood growth hormone (**A**) and IGF-1 (**B**) levels in female rat offspring on the day of weaning, and expression levels of IGF-1 mRNA (**C**), GH receptor mRNA (**D**), GH receptor protein (**E**) and miR-322 (**F**) in the liver were analyzed. To normalize each sample for RNA and miRNA content, GAPDH and U6 was used, respectively. To normalize each sample for protein content, beta actin was used. NBW: control rats (normal birth weight); LBW: low-birthweight rats due to calorie restriction; M: male rats (father); F: female rats (mother). One-way ANOVA followed by Turkey’s *post hoc* test was used. Data shown are means ± SEM.

**Figure 6:**
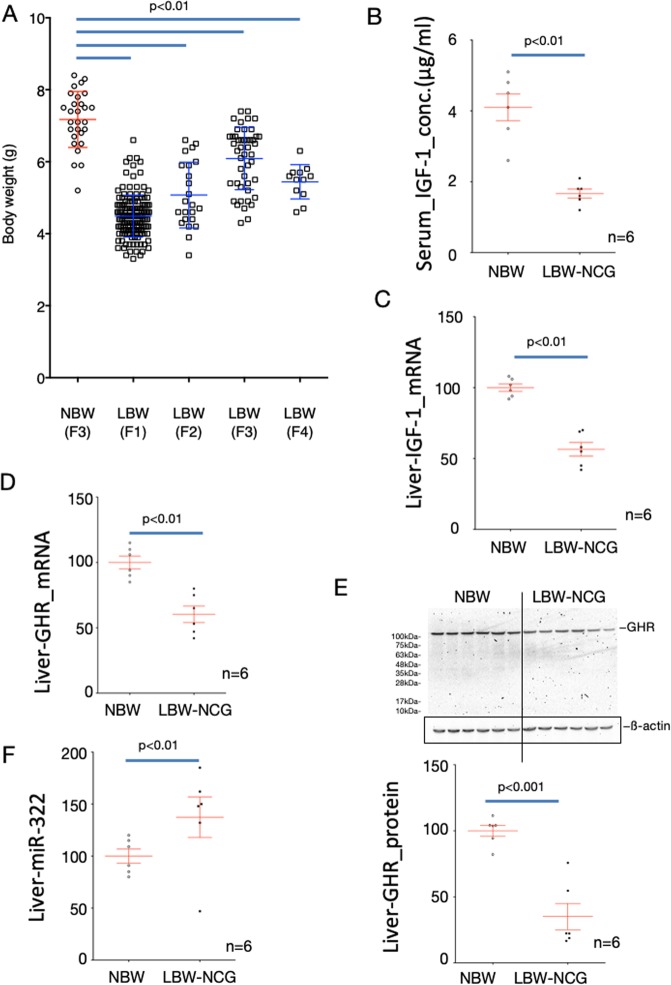
Birth weights of rat offspring after the next generation. We obtained offspring of the third and fourth generations from fathers and mothers of short-body-length, low-bodyweight rats whose growth did not catch up to normal (**A**). As in the second generation, pregnant dams were fed a standard diet *ad libitum*. The concentration of serum IGF-1 (**B**) and the expression levels of IGF-1 mRNA (**C**), GH receptor mRNA (**D**), GH receptor protein (**E**), and miR-322 (**F**) in the liver were analyzed. To normalize each sample for RNA and miRNA content, GAPDH and U6 were used, respectively. To normalize each sample for protein content, beta actin was used. NBW, control rats (normal birth weight); LBW, low birth weight rats produced by caloric restriction during pregnancy of the F_0_ generation; F_1_-F_4_ are the respective generations. One-way ANOVA followed by Turkey’s *post hoc* test (**A**) and unpaired *t* tests (**B–E**) were used. Data shown are means ± SEM.

Next, we investigated whether calorie restriction during pregnancy had an effect on the next generation in our rat model. Offspring were obtained from short-body-length and low-bodyweight parents who did not catch up in growth by the weaning stage. Although pregnant dams were given standard chow with free feeding without calorie restriction, the birth weights were significantly lower than those of the control (Fig. 4A). In addition, some of the F_2_ offspring of these low-birthweight rats failed to catch up until the weaning day and had short body lengths and low body weights (Fig. 4B,C). As with the F_1_ generation offspring, these short-body-length and low-body weight rats showed increased expression of miR-322 and decreased levels of GH receptor and IGF-1 expression in their livers without any change in plasma GH levels (Fig. 5). Next, we examined whether the influence of calorie restriction on the F_0_ generation during pregnancy was derived from the father or from the mother. If either the father or mother had a short body length and low body weight, the offspring had low birth weight and catch-up growth failed to occur. In addition, the expression levels of GH receptor and IGF-1 in the liver were decreased in any combination of these offspring (Fig. 5). When we examined the impact on subsequent generations, calorie restriction of F_0_ during pregnancy affected at least the F_4_ generation even though pregnant and lactating dams were given standard chow with free feeding (Fig. 6A). As with the F_2_ generation offspring, these low-birthweight F_3_ generation rats showed increased expression of miR-322 and decreased levels of GH receptor and IGF-1 expression in their livers (Fig. 6B–F).

### Suppression of GHR expression by miR-322

**Figure 7:**
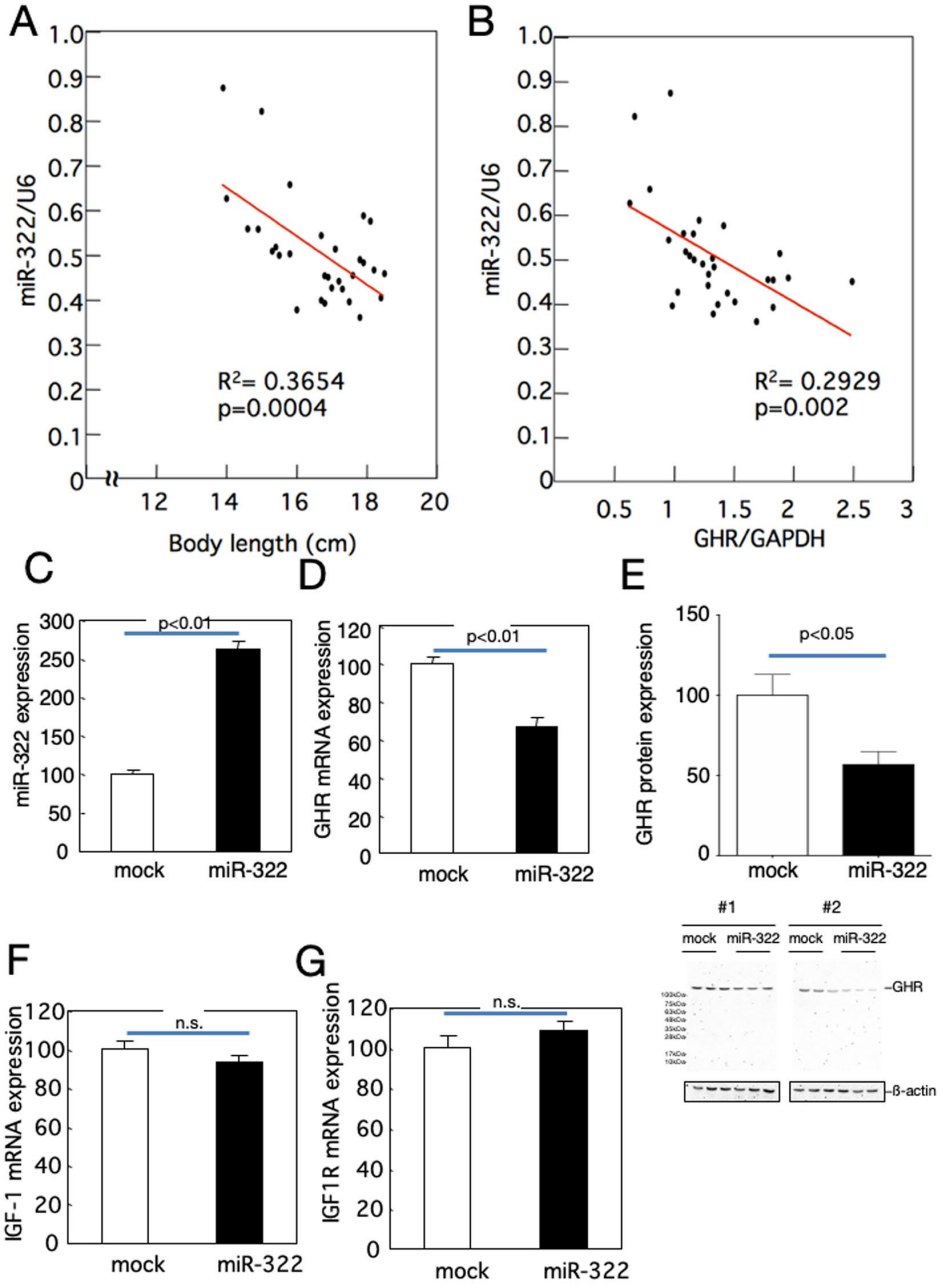
Suppression of GH receptor expression by miR-322 in primary cultured hepatocytes. The expression level of miR-322 in rat livers was correlated with body length (**A**) and the expression level of GH receptor (**B**). Decreased GH receptor mRNA (**D**) and protein (**E**) expression level by overexpression (**C**) of miR-322 in primary cultured hepatocytes, while the mRNA expressions of IGF-1 (**F**) and IGF1R (**G**) were not affected by miR-322 overexpression. To normalize each sample for RNA content, GAPDH or U6 snRNA were used. To normalize each sample for protein content, beta actin was used. Pearson correlation coefficient tests (**A**,**B**) and unpaired *t* tests (**C–G**) were used. Data shown are means ± SEM.

**Figure 8:**
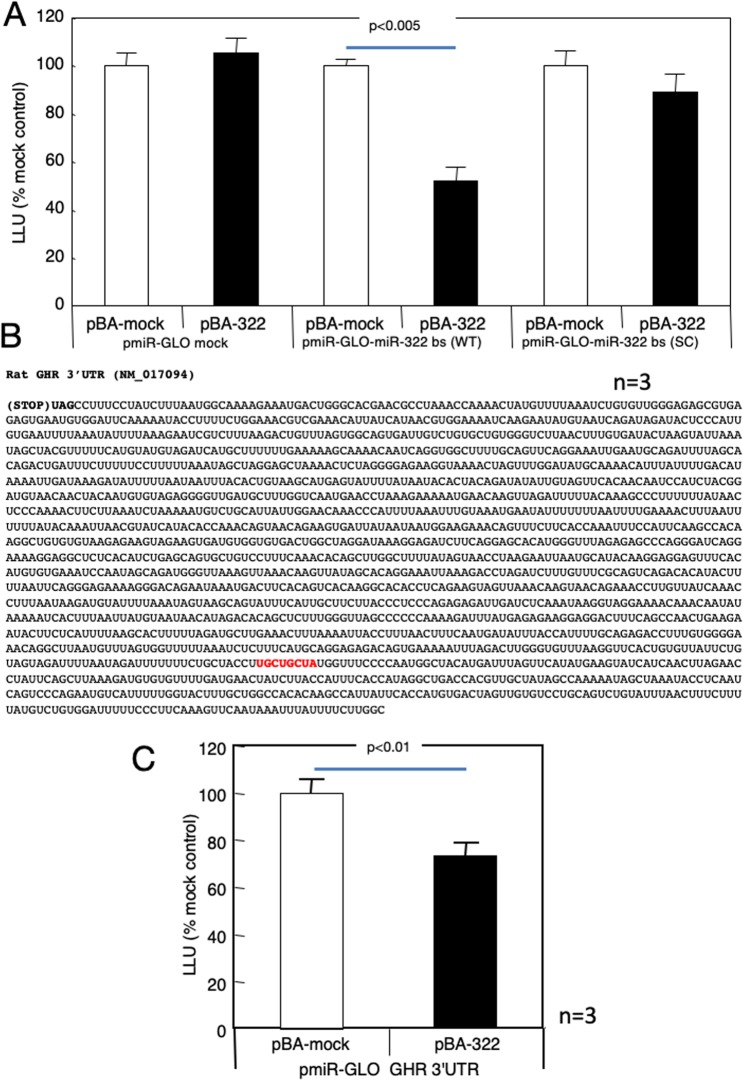
Reduction of luciferase activity by co-expression with miR-322. (**A**) Change in luciferase activity by co-expression with a luciferase construct in which the miR-322 binding region is mutated in HEK 293 cells or a construct in which the binding region is scrambled. (**B**) DNA sequence of rat GH receptor 3′-UTR. The binding region of miR-322 is indicated in red. (**C**) Changes in luciferase activity due to co-expression of a construct in which the full-length luciferase gene and the miR-322 forced expression construct are shown in (**B**). Unpaired *t* tests was used. Data shown are means ± SEM.

The expression level of miR-322 in the liver showed significant negative correlations with body length and with the expression level of GH receptor in the liver (Fig. 7A,B). Overexpression of miR-322 in primary cultured hepatocytes significantly decreased GH receptor expression levels (Fig. 7C–E), while IGF-1 and IGF1R expression levels were not affected (Fig. 7F,G). The luciferase activity decreased when the binding region of miR-322 was fused to the 3′-UTR of the luciferase gene and co-expressed with miR-322 in HEK293 cells (Fig. 8A). On the other hand, replacing the miR-322 binding region with a scramble mutation abolished the decrease in luciferase activity upon co-expression with miR322 (Fig. 8A). A sequence of the 3′-UTR of the GH receptor containing a binding region for miR-322 (shown in red letters in Fig. 8B) was fused to the 3′-end of luciferase. The luciferase activity decreased when the luciferase construct was co-expressed with miR-322 (Fig. 8C).

## Discussion

The results of this study revealed that calorie restriction during pregnancy not only delayed growth in the uterus but also, in part, delayed postnatal growth. It was also revealed that the growth retardation remained influential until at least the F_4_ generation. Furthermore, we found that the enhanced expression of miR-322 in the liver suppresses the expression of GH receptor, and the subsequent attenuation in growth hormone signaling decreases the expression and secretion of IGF-1. These results provide a mechanism for epidemiological phenomena such as the Dutch famine study and further clarify the endocrinological effects.

Growth disorder as a disease is an impairment due to a combination of genetics and environment. The disease afflicts approximately 10% of the population. Small for gestational age (SGA) short stature accounts for about 15% of cases, but the causes of many of the rest are not clear. Growth hormone receptor expression is strongly influenced by nutrients (especially protein intake)^25^. Although epigenetic modification of IGF-1 and IGF-BP3 have been reported^26,27^, there have been no reports of epigenetic modifications of GH receptors in low-birthweight infants who do not grow normally. We examined the DNA methylation changes in the 5′-UTR region of the GH receptor in the liver by pyrosequencing, but we could not find any difference between control and low-birthweight rats (71.0 and 66.3% in NBW vs. 73.0 and 67.0% in LBW-NCG, respectively). The GHR promoter, exon 1, and intron 1 are so long that it is possible that there was a change in the methylation of DNA elsewhere than where we examined. However, we concluded that there was little involvement of DNA methylation in the reduction of GHR expression, as no significant change was found in the analysis range using the Epigen DX algorithm.

In our rat model, growth hormone receptor levels were affected in the liver, but growth hormone levels were not affected. We hypothesized that hypothalamic-pituitary hormones in low-birthweight infants are not affected by malnutrition, during which brain weights and functions are protected, but instead the liver is a targeted as a trade-off and receives the brunt of the effects of low nutrition. We additionally found that the expression levels of GH receptors and IGF-1 in the liver decreased in the next generation. In addition, as a mechanism, increased expression of miR-322 in the liver occurred in LC-NCG rats, and the expression of miR-322 was significantly negatively correlated with body length and with GH receptor expression levels. There are several microRNAs that regulate growth hormone receptor expression in public databases, and several of these microRNAs have been reported in human cells^28^. We confirmed that all of them were expressed in the rat liver, but only miR-322 was found to be upregulated in LBW-NCG. Moreover, suppression of GH receptor expression by miR-322 was also demonstrated by overexpression experiments in primary cultured hepatocytes. Previous studies have reported that miR-322 reduces IGF-1 and IGF1R expression in several tissues^19–21^. However, in the overexpression experiment of miR-322 using our primary cultured hepatocytes, there was no significant change in the expression of IGF-1 or IGF1R. The details of the mechanism are unknown, but it seems that the gene expression regulation mechanism by miR-322 may have tissue specificity. In addition, the difference in overexpression was not due to cultured cells, and it cannot be denied that miR-322 suppresses IGF-1 in the rat liver. In any case, this is the first report that shows that miRNAs are involved in the regulation of gene expression in low-birthweight rats that did not experience catch-up growth. Moreover, the binding region of miR-322 in the 3′-UTR sequence of the GH receptor is conserved in many animal species, which implies its functional importance. Although we have not performed an analysis in humans, we expect that in humans as well as in rats, GH receptor expression is regulated by miR-322. One advantage of miRNA analysis is that it allows quantification in blood as well as in organs. In recent years, diagnosis by miRNA quantification in exosomes, called liquid biopsy, has been performed. In fact, blood miRNAs have begun to be measured as biomarkers for many diseases such as type 1 diabetes^29^, type 2 diabetes^30^, cardiovascular disease^31^, and others^32–35^. We also found that miR-322 is increased in blood exosomes of LC-NCG rats. We have not clarified whether increased expression in rat fetuses can predict subsequent growth, but we are preparing for future studies in human infants.

It has been reported that miR-322 is expressed not only in the liver, but also in many tissues such as skeletal muscle^16^, chondrocytes^17^, and vascular smooth muscle^18^. However, the detailed role of transcription factors in the regulation of miRNA expression are still unclear. It has been shown that the transcription factor FAM3B negatively regulates miR-322 expression during myocyte differentiation, which affects the expression of SETD3^16^. This indicates that there are two parallel regulatory cascades in which transcription factors regulate miR-322 fate, thereby regulating the expression of SETD3 and ultimately determining skeletal muscle differentiation. Thus, in skeletal muscle, although regulation of miR-322 expression has been partially elucidated, transcriptional regulation in the liver is still unknown. Also, the involvement of DNA methylation in miRNA expression has not been elucidated. Recently, it was reported that differentially expressed miRNAs exist in several miRNA clusters, including the DLK1-DIO3 genomic imprint cluster in large offspring syndrome and Beckwith-Wiedemann syndrome, and hypermethylation of DNA downregulates miRNAs in DLK1-DIO3 in these diseases^36^. We still do not know why the expression of miR-322 was elevated in LC-NCG rats. In the F_1_ generation, methylation was weakly but significantly decreased at two CpG sites in the 5′-UTR of miR-322 (37.9 and 55.3% in NBW vs. 21.7 and 41.9% in LBW-NCG, respectively, n = 3). However, despite the increased expression of miR-322 in the livers of the F_2_ generation, there was no change in the profile of the CpG site where methylation was increased at two sites in the F_1_ generation of LBW-NCG (45.0 and 67.0% in NBW vs. 51.6 and 60.3% in LBW-NCG, respectively, n = 3). It is possible that other methylation sites or mechanisms other than DNA methylation may be involved. It is known that the expression of miRNAs changes by epigenetic modification, but miRNAs themselves cause epigenetic modification^24^. It has been reported that miR-139 is epigenetically silenced by histone H3 lysine 27 trimethylation (H3K27me3) of its host gene PDE2A in non-small cell lung cancer, and that this process is independent of promoter DNA methylation^37^. We need detailed investigations into the mechanism of increased expression of miR-322, including histone methylation as well as DNA methylation in low-birthweight rats. In addition, we have revealed that changes in nutritional status and miR-322 expression levels are at least not affected by glucose concentration, but other mechanisms have yet to be elucidated. It has been reported that the expression level of GH receptor varies depending on the amount of protein intake^25^, but details of the changes in miR-322 and GH receptor expression levels due to changes in nutritional status are still unclear. In the future, we will analyze the liver metabolome to provide more details.

Besides the liver, GH receptor and IGF-1 are expressed in cartilage, and it has been reported that there is no difference in growth even if the GH receptor or IGF-1 are knocked-out specifically in the liver^38,39^. In our preliminary experiments, we found that the expression of the GH receptor in the fibula cartilage of low-birthweight rats that failed to catch up was reduced. We detected miR-322 from exosomes in the blood, but it is not clear whether the exosomes were from the liver or not. Since exosomes containing miR-322 were detected in the supernatants of primary hepatocyte cultures, it is certain that the liver releases miR-322-containing exosomes into the blood. Because exosomes are generally released into the blood and then taken up by other cells, it is possible that liver-derived exosomes, including those with miR-322, may act on cartilage. Unfortunately, we were unable to properly quantify miR-322 in cartilage, but it has been reported that miR-322 is also expressed in cartilage^17^, so it is also possible that the expression of miR-322 is increased in the cartilage of low-birthweight rats. We plan to investigate whether there is a difference in the regulatory mechanism of expression in the liver and epiphyseal cartilage in the future.

In our model, there was no significant difference in the length and weight of the pups when comparing only the father alone or the mother alone and both parents. The reason why there was no difference between one parent and both parents is unknown. It has been reported that the pathogenesis of human fetal growth restriction is due to the increased level of miR-424 in placental tissue and mediation by low levels of FGFR1 and MEK1^40^. In our model, we confirmed by preliminary experiments that the expression of placental IGF-2 is decreased in LBW-NCG rats, but the relationship between miR-424 and miR-322 is unknown. The failure of catch-up growth in LBW-NCG littermates may be due to elevated miR-322 levels in the F_1_ placenta, but the detailed mechanism is unclear.

Our results show that one cause of the obstruction of catch-up growth in low-birthweight rats caused by fetal malnutrition is a decrease in GH receptor expression and a decrease in IGF-1 secretion due to increased expression of miR-322 in the liver. Moreover, calorie restriction during pregnancy affected birth weights at least to the F_4_ generation, even though dams were given standard chow with free feeding during pregnancy and lactation.

## Materials and Methods

### Animals

Wistar rats were maintained at 23 ± 2 °C with a 12:12-h light-dark cycle (lights on at 0800 h, off at 2000 h). They were allowed *ad libitum* access to laboratory chow and distilled water. All experimental procedures were reviewed and approved by the Laboratory Animals Ethics Review Committee of Nippon Medical School^41^. All experiments were performed in accordance with relevant guidelines and regulations.

Twenty proestrus female rats (age, 9 weeks) were mated with normal male rats. Dams (F_0_ generation) were housed individually with free access to water, and were divided into two groups: low carbohydrate and calorie restricted diet (LC) dams were restricted in their calorie intake to 60% of the control group (D08021202, Research Diet Inc., New Brunswick, NJ), while control dams freely accessed food during the entire gestational period. The number of births was adjusted to 10~12 per mother rat, and the mother rats after birth were given standard chow by free-feeding in both groups. Offspring with birth weights of 5.7 g (mean-2SD of the control) or more were marked and excluded at weaning. Offspring (F_1_ generation) from LC dams and controls were weighed on their birth dates, and the mother rats after delivery received standard chow with free feeding. Next-generation (F_2_) offspring were obtained using short-length and low-bodyweight male and female rats. Short-length and low-body-weight maternal rats after mating were fed a standard chow with free feeding. In the same way, we obtained F_3_ and F_4_ generation offspring.

### Primary cultures of hepatocytes

Primary cultured stem cells were purchased from Takara Bio (Shiga, Japan) and cultured with DMEM/F10 HAM culture medium (Sigma-Aldrich Co., St. Louis, MO) supplemented with 10%FBS in a 37 °C humidified 95% air/5% CO_2_ environment. The cells were cultured for 4 days until attached, and then overexpression experiments were performed. After 2 days of incubation, cells were collected and extracted their RNA and protein.

### miRNA overexpression in primary hepatocytes

The microRNA overexpression experiment used the same method we previously performed in the miR-449a overexpression experiment^42^. Briefly, each miRNA overexpression vector (pmr-mCherry-rno-miR-322a or pmr-mCherry-mock) was purchased from Takara Bio. DNA fragments, which included the miRNA, were then cloned into the pAxcwit2 cosmid vector (Takara Bio) (pAx-miR322 or pAx-mock). These cosmids were used according to the manufacturer’s instructions (Takara Bio) and the COS-TPC method to construct each recombinant adenovirus. Primary hepatocytes were infected with adenovirus by adding the viruses to culture media at a 10^6^ MOI. To check whether a recombinant adenovirus successfully infected the cells, a fluorescent microscope was used to assess red fluorescence 48 h after transfection.

### Exosome purification

Exosome extraction was performed using the same method as previously used^41^. Serum samples from trunk blood or culture media were centrifuged at 3,000 × *g* and 4 °C for 30 min to remove debris. Each 250 μl sample (serum or culture media) was mixed with ExoQuick exosome precipitation solution (System Bioscience, Inc., Mountain View, CA) according to the manufacturer’s instruction. Purified exosome samples were resuspended in ultrapure water and were then used for miRNA quantification.

### RNA extraction and real-time RT-PCR analysis

RNA extraction and real-time RT-PCR were performed using the same method as previously used^41^. Total RNA was extracted from rat liver and primary-cultured hepatocytes using RNAiso Plus (Takara Bio). For miRNA expression analysis, first strand cDNA was synthesized using 1 μg of denatured total RNA at 37 °C for 1 h; reactions were terminated at 85 °C for 5 min with the Mir-X^®^ miRNA First-Strand Synthesis and SYBR^®^ qRT-PCR kits (Clontech Laboratories Inc., Mountain View, CA). For mRNA expression analysis, 0.5 μg of denatured total RNA and PrimeScript^®^ RT reagent kit with gDNA Eraser (Takara Bio) were used to synthesize first strand cDNA; reactions were incubated at 37 °C for 15 min, 84 °C for 5 s, and finally 4 °C for 5 min. Each PCR proceeded via denaturation at 94 °C for 5 s and annealing-extension at 60 °C for 30 s for 40 cycles; SYBR premix Ex Taq (Takara Bio) and specific primers for rat GHR, IGF-1, IGF1R, and GAPDH (Takara Bio, RA009268, RA028844, RA026001, and RA015380, respectively) were used. To normalize each sample for RNA content, GAPDH, a house-keeping gene, or U6 small nuclear RNA (Clontech Laboratories, Inc.) were used for mRNA and miRNA expression analyses, respectively.

### Hormone assay

Plasma GH and IGF-1 were analyzed by a Rat GH ELISA kit (Shibayagi, Co, Ltd., Gunma, Japan) and Mouse/Rat IGF-1 Immunoassay kit (R&D systems, Minneapolis, MN), respectively. For liver tissue content of IGF-1, livers were lysed with PBS containing the Complete Proteinase Inhibitor Cocktail (Roche). Lysates were subjected to centrifugation to remove debris, the protein concentrations in each supernatant were measured, and then supernatants were used for ELISA.

### Western blotting

Protein samples from rat liver or miR-322 overexpressing-cells were extracted using complete lysis-M (Roche, Mannheim, Germany). The protein concentrations of lysate samples were determined using the Pierce 660 nm Protein assay (Thermo Scientific, Rockford, IL). Each 40 µg protein was electrophoresed on a 4–15% gradient Criterion TGX gel and transferred to a nitrocellulose membrane. The transfer membranes were blocked with 4% skim milk and then incubated with an anti-GHR antibody (1: 200, ab134078, abcam, Cambridge UK) for 1 hour at room temperature followed by an additional overnight incubation at 4 °C. The transfer membranes were washed with TBS-T, and further incubated with HRP-labeled anti-rabbit IgG (1: 2000, Jackson Immunoresearch, West Grove, PA) for 1 hour at room temperature. The signals were detected using SuperSignal West Dura extended duration substrate (Thermo Scientific). The membranes after detection were stripped of the antibody using Restore plus western blot stripping buffer (Thermo Scientific). Then, signals were detected again using THE^TM^ [HRP] -labeled beta actin antibody (1:1,000 A00730-40, GeneScript, Pitcataway, NJ). The expression levels of GHR were quantified by correcting the GHR signal with the ß-actin signal.

### The 3′-UTR assay

The 3′-UTR assay used the same method we previously performed in the miR-449a overexpression experiment^42^. Briefly, RT-PCR using a forward primer (tagcctttcctatcttttaatgg) and reverse primer (gccaagaaaataaatttattg) was used to amplify a segment of the GHR 3′-UTR sequence (NM_017094) that contained an miR-322 binding sequence from normal rat liver RNA; this amplified segment was designated GHR 3′-UTR. We also synthesized a negative control oligonucleotide with a mutant miR-322 target site. TGCTGCTA was mutated to GCTGCTAT; additionally, the In-Fusion cloning kit (Takara Bio) was used to generate a mutant variant of the GHR binding site (bs). This variant was designated GHR bs (sc). The GHR bs fragment and the corresponding negative control variant were subcloned into pmir-GLO vector (Promega, Madison, WI) to generate pmir-GLO-GHR bs (wt) and pmiR-GLO-GHR bs (sc), respectively. HEK293 cells were seeded in 60-mm dishes, and Multifectam (Promega) was used to cotransfect cells with a target plasmid (2.5 μg) (pmir-GLO-GHR 3′-UTR [wt], pmir-GLO-GHR 3′-UTR [sc] or pmir-GLO mock) and a miRNA plasmid (pmr-mCherry-miR-322 or pmR-mCherry mock plasmid). Cells were collected 48 h after transfection, and the dual-luciferase reporter assay system (Promega) was used according to the manufacturer’s instructions to measure enzyme activity.

### Statistical analyses

Paired *t* tests, unpaired *t* tests, Pearson correlation coefficient tests, or a one-way analysis of variance (ANOVA) followed by Turkey’s *post hoc* test for multiple comparisons were used for each statistical analysis; Prism 5.0 software (GraphPad Software, Inc., La Jolla, CA) was used for all calculations. Each study involving primary cultured hepatocytes and HEK293 cells was performed at least twice using the same protocol to confirm consistency. Real-time RT-PCR and Western blot data were expressed as percent ± SEM with 100 as the control. p < 0.05 was considered statistically significant.
